# Editorial: Plasmonic Technologies for Bioanalytical Applications

**DOI:** 10.3389/fchem.2019.00865

**Published:** 2019-12-13

**Authors:** Sebastian Wachsmann-Hogiu, Zachary J. Smith, Mehmet Kahraman

**Affiliations:** ^1^Department of Bioengineering, McGill University, Montreal, QC, Canada; ^2^Department of Precision Machinery and Precision Instrumentation, University of Science and Technology of China, Hefei, China; ^3^Department of Chemistry, Gaziantep University, Gaziantep, Turkey

**Keywords:** plasmonics, bioanalytical, medical diagnosis, extracellular vesicles, plasmonic materials

Bioanalytical technologies that provide quantitative information about bioanalytes of interest in biological systems are becoming widespread in biomedical applications, environment monitoring, and food quality and safety. Among recent developments, plasmonic-based technologies made significant strides toward real life applications. They are based on surface plasmons, which are collective oscillations of conductive electrons on noble metal film or nanoparticle surfaces induced by electromagnetic radiation at the metal-dielectric interface. Plasmonic nanostructures have the ability to control and manipulate visible light at the nanometer scale and have been used to create plasmonic filters, wave-guides, nanoscopic light sources, and other devices with unique or improved properties. In addition, plasmonics has helped further our understanding of the interaction of molecules with nanostructures and has been used in biosensing applications. Their low ohmic and optical losses, chemical activity, tunable optical properties, specific response to incident light, and the ability to enhance signals such as fluorescence and Raman scattering, have been exploited in biomedical applications. Specific plasmonic-based technologies include nanoscale optical absorption spectroscopy, surface plasmon resonance (SPR), localized surface plasmon resonance (LSPR), surface-enhanced Raman scattering spectroscopy (SERS), tip-enhanced Raman spectroscopy (TERS), surface-enhanced infrared absorption spectroscopy (SEIRAS), single molecule plasmonics (SMP), and chiral plasmonics (CP). This special issue is needed for chemists, biologists, and materials scientists who work in the area of plasmonics applications in bioanalytical sensing.

As described above, the potential utility of plasmonics as a bioanalytical sensing technique is already well-acknowledged. Our collection illustrates the expanding frontiers of plasmon-based bioanalysis through a series of timely articles. Rojalin et al. show how nanoplasmonic methods such as SPR, SERS, and other emerging techniques can be used to achieve exquisite characterization of extracellular vesicles (EVs). EVs are nano-scale, membrane-bound vesicles containing protein and active RNA and DNA cargo that are released by biological cells into the extracellular space, and form an important part of cell-cell communications (Raposo and Stoorvogel, 2013). As they are critical to cell function, and are increasingly implicated in a wide variety of diseases, including cancer, they are a topic of intense study. However, their nanometer size and relatively low abundance makes them difficult to assay using conventional methods. Using nanoplasmonics to study the chemical profile of small numbers (down to single EVs; Lee et al., 2015; Stremersch et al., 2016) has the potential to greatly improve our understanding of EV function and heterogeneity.

However, despite the appealing application prospects of plasmonic sensing, with the exception of SPR, the majority of plasmonic methods have yet to make the transition to commonplace, commercial analytical assays. Intense research is still necessary to further our fundamental understanding of plasmonic methods, which by their very nature arise from complex nanoscale interactions, as well as refine methods to improve the repeatability and cost-effectiveness of plasmonic approaches.

In our collection, we are pleased to have one article describing just such steps forward in our understanding. Szekeres and Kneipp demonstrate how protein-induced nanoparticle aggregation alters the SERS spectrum in a concentration-dependent manner. In particular, in homogeneous solutions of proteins with concentrations resembling those in a living cell, a lack of aggregation was observed, owing to the protein completely coating the spheres and preventing aggregation. Thus, the strong and heterogeneous enhancement produced by nanoparticle aggregation within cells is likely due to protein concentration heterogeneities and other active effects within cells themselves.

Finally, Campu et al. demonstrate a unique and attractive multi-plasmonic-mode paper-based analytical device for point-of-need diagnostic applications. By carefully constructing a gold metal bipyramid system, which could be simply drawn onto a paper surface with a standard office pen, Campu et al. were able to obtain LSPR shifts, alterations in the SERS spectrum, and alterations in metal-enhanced fluorescence, from the same streptavidin-biotin binding process. The relative ease of fabrication and the multimodal nature of the system represent an intriguing advance for field-portable diagnostics, and represents an important step in the emerging field of paper-based analytical devices (Martinez et al., 2010; Lisowski and Zarzycki, 2013).

The use of plasmonics for bioanalytical applications has increased dramatically in recent years. While significant steps are made to increase the sensitivity and specificity of detection, other factors must be considered for the test to have value beyond a simple academic exercise (Drain et al., 2014), such as cost, usability, manufacturability, reproducibility, stability. In particular reproducibility of plasmonic biosensors (which is related to the non-uniformity of hot spots) has been a problem that hampered their use in biomedical applications. On the other hand, the potential for use in food-related and environmental monitoring has not been exhaustively explored yet. In order to push the research field forward, advances must take place both in the fundamental understanding of plasmonics, and in how to best tailor that understanding in service of practical assays that could be performed by non-experts, at the point of need. Designing materials and structures with improved plasmonic properties for specific applications will be most important.

## Author Contributions

All authors listed have made a substantial, direct and intellectual contribution to the work, and approved it for publication.

### Conflict of Interest

The authors declare that the research was conducted in the absence of any commercial or financial relationships that could be construed as a potential conflict of interest.
